# Gadolinium-labelled iron/iron oxide core/shell nanoparticles as *T*_1_–*T*_2_ contrast agent for magnetic resonance imaging†

**DOI:** 10.1039/c8ra04530e

**Published:** 2018-07-26

**Authors:** Kaili Wang, Lu An, Qiwei Tian, Jiaomin Lin, Shiping Yang

**Affiliations:** The Key Laboratory of Resource Chemistry of the Ministry of Education, The Shanghai Key Laboratory of Rare Earth Functional Materials, The Shanghai Municipal Education Committee Key Laboratory of Molecular Imaging Probes and Sensors, Shanghai Normal University Shanghai 200234 China qiweitian@shnu.edu.cn shipingy@shnu.edu.cn

## Abstract

Magnetic resonance imaging (MRI) is indispensable and powerful in modern clinical diagnosis and has some advantages such as non-invasiveness and high penetration depth. Furthermore, dual *T*_1_–*T*_2_ MR imaging has attracted crucial interest as it can decrease the risk of pseudo-positive signals in diagnosing lesions. And it's worth nothing that the dual-mode MR imaging displays a vital platform to provide relatively comprehensive diagnosis information and receive accurate results. Herein, we report a dual *T*_1_–*T*_2_ MR imaging contrast agent (CA) grounded on the iron/iron oxide core/shell nanomaterials conjugated with gadolinium chelate. The Gd-labeled Fe@Fe_3_O_4_ NPs reveal the feasibility to utilize them to serve as a dual *T*_1_–*T*_2_ MR imaging CA, and the relaxivity results in a 0.5 T MR system showed a longitudinal relaxivity value (*r*_1_) and transverse relaxivity value (*r*_2_) of 7.2 mM^−1^ s^−1^ and 109.4 mM^−1^ s^−1^, respectively. The MTT results demonstrate the Gd-labeled Fe@Fe_3_O_4_ NPs have no obvious cytotoxicity and a good compatibility. The *in vitro* and *in vivo* MRI generated a brighter effect and darkening in *T*_1_-weighted MR imaging and *T*_2_-weighted images, respectively. The results clearly indicate that Gd-labeled Fe@Fe_3_O_4_ NPs have potential as a magnetic resonance imaging contrast reagent.

## Abstract 2

Magnetic resonance imaging (MRI) is indispensable and powerful in modern clinical diagnosis and has some advantages such as non-invasiveness and high penetration depth.

## Graphical abstract

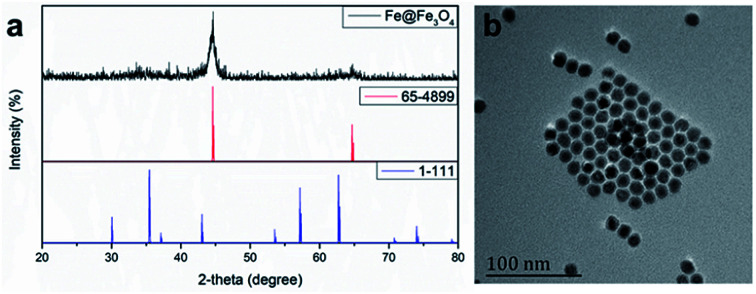

## 1. Introduction

Cancer has become a predominant cause of death across the world and seriously threatens human health.^1,2^ Hence, exact diagnosis of cancer becomes extremely important. During the past few decades, the representative imaging modalities in either preclinical research or clinical settings are magnetic resonance imaging (MRI), computed tomography (CT), positron emission tomography (PET), optical fluorescence imaging (FI), single-photon emission computed tomography (SPECT), ultrasound (US), and photoacoustic imaging (PA).^3–5^ Even so, when we need precise small animal imaging, such a single imaging mode is defective and more challenges arise in complex environments.^6^ To settle this problem and achieve accurate molecular imaging, a multi-mode imaging technique is one of the promising ways.^7^ Therefore, multifunctional CAs are usually indispensable as the complementary information of different imaging modes can be obtained at one time.^8^

As one of the most excellent modern clinical imaging techniques, MRI has become an extremely important part due to its noninvasive character, high soft tissue contrast, large penetration depth and outstanding spatial resolution.^9–11^ Therefore, MRI integrated multi-mode imaging techniques including MR-PET/SPECT, MR-Optical, MR-Ultrasound and MR-Photoacoustic were investigated widely.^12–16^ However, it is worth noting that these multi-mode imaging techniques must be performed on different imaging devices, which cost much more. Recently, multi-mode imaging carried out on one device, such as the *T*_1_–*T*_2_ dual-weighted MRI, has attracted more attention.^17–20^ Furthermore, the difficulties of image matching in the multiple imaging instruments could be eliminated when a single imaging system was selected, in other words, the accuracy of tumor detection was improved.^21,22^ Thus, it is necessary to design and synthesis of the dual *T*_1_–*T*_2_ MRI CAs for realizing dual-modal imaging on a single instrument.^6,11,23^ To date, a great deal of work in effects have already been carried out to prepare *T*_1_–*T*_2_ dual-weighted CAs for MRI *via* the combination of iron materials with Gd elements. For example, Liang *et al.* prepared the core/shell Fe_3_O_4_/Gd_2_O_3_ nanocubes and put it into use for heightened contrast effect.^24^ Zhang *et al.* applied Fe_3_O_4_ as a core, SiO_2_ as a separating layer, Gd_2_O(CO_3_)_2_ as the outer shell to synthesis core/shell/shell nanoparticles and explored the potential of images.^25,26^ Chen *et al.* used hydrophobic interaction between gadolinium with iron oxide to gain a new nanocrystal (GdIO). This novel GdIO nanocluster showed an important MRI monitoring capability.^27^

**Scheme 1:**
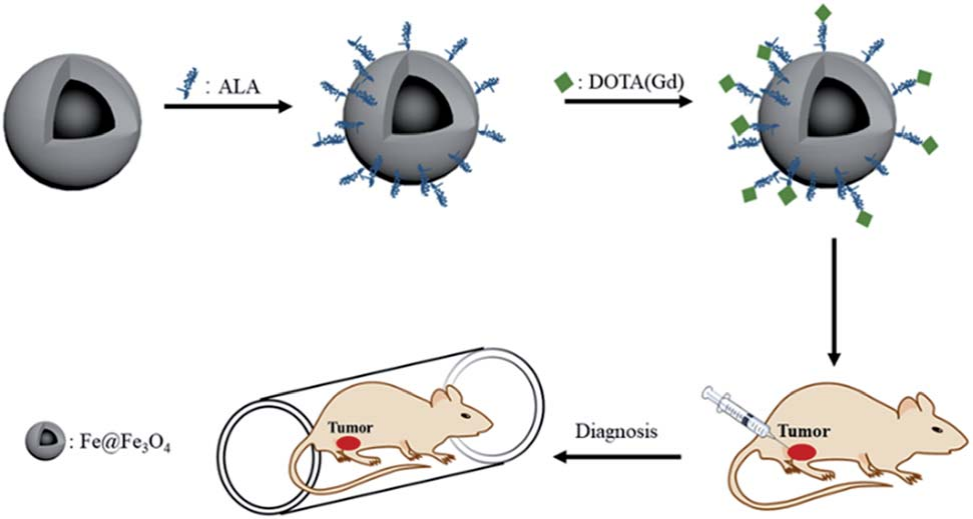
Schematic of the MRI of the DOTA(Gd)-Fe@Fe_3_O_4_ NPs.

Besides, Long *et al.* also reported an underlying *T*_1_–*T*_2_ MRI probe of gadolinium-labelled Fe_3_O_4_ nanoparticles but did not further used especially *in vivo*.^28^ Inspired by this, in order to get even more in-depth imaging information and expand the application of the probe, we conjugated the Gd chelates onto Fe@Fe_3_O_4_ nanoparticles to create a platform for dual modal detection. Because iron has a good biocompatibility, so Fe@Fe_3_O_4_ core/shell NPs have been widely treated on *T*_2_-weight MRI CAs. Our group have developed several *T*_2_-weight MRI guided therapy agents based on the excellent *T*_2_-weight MRI properties of the Fe@Fe_3_O_4_ core/shell NPs. Gadolinium(iii) containing MRI CAs are the commercial and most commonly used *T*_1_-MRI agents. The most used injection reagent of the Gd based *T*_1_-MRI agents is a chelated compound due to the toxic of the free solubilized aqueous Gd ion.^29^ Thus, it offers an opportunity for the Gd chelated compound to undergo ligand exchange with Fe@Fe_3_O_4_ core/shell NPs, forming a dual *T*_1_–*T*_2_ MRI CA. Hence, to expand the application of CAs in MRI and increase the sensitivity detection of lesion sites, we fabricate a dual-functional Gd-labeled Fe@Fe_3_O_4_ NPs for dual *T*_1_–*T*_2_ MR images. The formation process of NPs consisted of three procedures (Scheme 1). Firstly, the Fe@Fe_3_O_4_, capping with oleic and oleylamine, was formed through the thermal decomposition approach. In this procedure, Fe(CO)_5_ was utilized as a precursor. Secondly, alendronate-coated Fe@Fe_3_O_4_ was prepared *via* the ligand exchange based on the strong coordination ability of the Fe and phosphonic acid groups by mixing the obtained Fe@Fe_3_O_4_ NPs and alendronate solution. Then, the acquired alendronate-coated Fe@Fe_3_O_4_ NPs were combined with 1,4,7,10-tetraazacyclododecane-1,4,7,10-tetraacetic acid (DOTA), one of the typical Gd chelating agents, by activating with 3-(3-dimethylaminopropyl)-1-ethylcarbodiimide hydrochloride/*N*-hydroxysuccinimide (EDC/NHS) method. After purification by removing the unreacted reagent, Gd^3+^ was added to get DOTA(Gd)-Fe@Fe_3_O_4_ NPs by chelating with DOTA. The formed multifunctional DOTA(Gd)-Fe@Fe_3_O_4_ NPs were characterized by transmission electron microscopy (TEM), X-ray diffraction (XRD), Fourier transform infrared spectroscopy (FTIR), relaxivity measurements and MRI. At the same time, their effect of tumor diagnosis on 4T1 cells *in vitro* and *in vivo* were further evaluated. Our results indicated that DOTA(Gd)-Fe@Fe_3_O_4_ NPs played an important role in tumor diagnosis.

## 2. Experimental section

### 2.1. Apparatus and reagents

Oleic acid, 1-octadecene, oleylamine, 3-(3-dimethylaminopropyl)-1-ethylcarbodiimide hydrochloride (EDC) and *N*-hydroxysuccinimide (NHS) were supplied from Sigma-Aldrich. GdCl_3_·6H_2_O, alendronate sodium (ALA) and DOTA were provided from Adamas-beta. Fe(CO)_5_ was supplied from Development of Beijing Chemical Technology Co., Ltd. Branch. All the chemicals were used without further purify.

XRD of the Fe@Fe_3_O_4_ was obtained on a Rigaku DMAX 2000 diffractometer (Cu/Kα radiation, 40 kV, 40 mA, scan range 20–80°). TEM images were measured on a JEOL JEM-2010 microscope with the accelerating voltage in 200 kV. The dynamic light scattering (DLS) and surface potential were collected utilizing a Malvern Zetasizer Nano ZS model ZEN3600 (Worcestershire, U.K.). FTIR spectra were captured by a Nicolet Avatar 370 FTIR spectrometer. The concentration of the metal in the samples was collected by inductively coupled plasma atomic emission spectroscopy (ICP-AES). The hysteresis loops were performed in a superconducting quantum interference device (SQUID) from Lake Shore.

### 2.2. Synthesis of ALA coated Fe@Fe_3_O_4_ NPs

Firstly, as previously reported, 12 nm Fe@Fe_3_O_4_ samples were prepared.^30^ Then, the ALA coated Fe@Fe_3_O_4_ was prepared by a ligand exchange method. The details as follow: 100 mg ALA were dispersed in 15 mL mixture of water and DMF (v/v = 2 : 1) in single-necked flask. Then, 8 mL oleic acid-coated NPs in DMF (3 mg mL^−1^) and 0.4 mL KOH (1 M) was added to the above mixture. After shaking for 12 hours at room temperature, the ALA coated Fe@Fe_3_O_4_ NPs was captured by centrifugation and washed with acetone *via* several times. At last, nanoparticles were dissolved in 10 mL water for next utilize.

### 2.3. Preparation of DOTA-Fe@Fe_3_O_4_ NPs

In details, 10 mL of double distilled water containing NHS (4.268 mg) and EDC (4.740 mg) was mixed with DOTA (10 mg, 5 mL) underneath magnetic stirring. After 2 h, the DOTA which activated was appended into ALA coated Fe@Fe_3_O_4_ NPs water dispersion, and this mixture was reacted for overnight. After collecting by centrifugation and washed with acetone, the acquired product was dispersed in sodium acetate buffer (0.2 M, pH = 6.5) for subsequent use.

### 2.4. Preparation of DOTA(Gd)-Fe@Fe_3_O_4_ NPs

In a typical process, a GdCl_3_·6H_2_O aqueous solution (10 mg, 5 mL) was appended to the prepared DOTA-Fe@Fe_3_O_4_ sodium acetate buffer solution dispersion. After stirring for 10 h, DOTA(Gd)-Fe@Fe_3_O_4_ NPs were obtained by centrifugal separation and washed with acetone *via* several times. At last, the obtained DOTA(Gd)-Fe@Fe_3_O_4_ NPs were dispersed in distilled water and preserved in 4 °C before utilization.

### 2.5. Cytotoxicity assay

Methyl thiazolyl tetrazolium (MTT) measurement was utilized to assess the cytotoxicity of the DOTA(Gd)-Fe@Fe_3_O_4_ NPs by means of 4T1 cell line. 4T1 cells were seeded into 96-well culture plates (5 × 10^4^ cells per well) in DMEM assisted by 1% penicillin-streptomycin and 10% fetal bovine serum at 37 °C under 5% CO_2_. After being cultured for 12 h, the DOTA(Gd)-Fe@Fe_3_O_4_ NPs with different concentrations (0, 10, 20, 50, 100, 200 μg mL^−1^ dispersed in DMEM) were added to culture the cells for a further 12 or 24 h, respectively. Thereafter, the cells were incubated for another 4 h when a certain amount of MTT solution (20 μL 5 mg mL^−1^) was appended to each well under a similar condition. After that, with the adding of DMSO (150 μL per well), the blue formazan crystals were captured after shaking (150 rpm, 5 min). At last, the absorption was acquired by microplate reader.

### 2.6. Relaxivity measurements in solution

The relaxation characteristics of both DOTA(Gd)-Fe@Fe_3_O_4_ NPs and ALA coated Fe@Fe_3_O_4_ NPs at various iron concentrations were test to evaluate the dual-model MR performance. All nanoparticles were dispersed in distilled water and carried out on the 0.5T NMI20 Analyst (Shanghai Niumag Electronic Technology Co., Ltd.). As to *T*_1_-weighted relaxation time, it was collected by a spin-echo sequence, and parameters were as follows: TW = 10 000 ms, NS = 2, *P*1 = 10.48 μs, SW = 100 KHz, RFD = 0.020 ms, RG1 = 20.0 db, DRG1 = 3, TD = 1024. For *T*_2_-weighted relaxation time, TW/TE = 12 000/1.5 ms, SF = 18 MHz, *P*2 = 20.00 μs, NECH = 15 000. The other parameters are similar to *T*_1_-weighted MRI. The acquired fitted curves for 1/*T* (s^−1^) against the Fe concentration (mM) were analyzed to calculate the relaxivity values of *r*.

### 2.7. MRI in cells

The *in vitro* dual-model MRI performance of the prepared DOTA(Gd)-Fe@Fe_3_O_4_ NPs was test by incubating the DOTA(Gd)-Fe@Fe_3_O_4_ NPs with 4T1 cells on 0.5T NMI20 Analyst. In details, 4T1 cells were seeded in a 6-well plate at a cell density of 1 × 10^6^ cells per mL for 12 h. Then, the cells were incubated with DOTA(Gd)-Fe@Fe_3_O_4_ NPs for 4 h. After that, the cells were resuspended in xanthan gum (1 × 10^6^ cells per well) after washed with PBS buffer solution for several times. Given the above, the preparation of MRI in cells was completed. For *T*_1_-weighted MRI, parameters were determined as follows: *D*0 = 500 ms, *D*4 = *D*5 = 50 ms, RFA1 = 5.5%, RFA2 = 9.5%, RP1Count = 2, RP2Count = 128. For *T*_2_-weighted MRI, *D*0 = 5500 ms, *D*4 = *D*5 = 200 ms. The others of parameters were the same.

### 2.8. MRI *in vivo*

The *in vivo* dual-model MRI was performed by a 0.5 T system (MiniMR-60; Shanghai Niumag Electronic Technology Co., Ltd.). The prepared DOTA(Gd)-Fe@Fe_3_O_4_ NPs was intertumoral injected in 4T1 tumor mouse. Noted, animal experiments were approved by the animal ethics committee of Shanghai Normal University and in strict accordance with the policy of the Institutional Animal Care and Use Committee. Firstly, the 4T1 tumor bearing mice model was prepared by inoculating subcutaneously with 4T1 cells (3 × 10^7^ cells per mL) for female BALB/c mice (6 weeks). After diameter of tumor reached ∼1.2 cm, the DOTA(Gd)-Fe@Fe_3_O_4_ NPs (50 μL, 2 mg mL^−1^) was injected into BALB/c mice *via* intertumoral injection. As to MRI *in vivo*, experiments were performed on the 0.5 T MRI system after injected for 30 min. For *T*_1_-weighted MRI, a conventional spin-echo sequence was utilized, the parameters were as follows: TR/TE = 300/12 ms, 192 × 256 matrices, 80 × 80 mm field of view, *P*1 = 5.3, NS = 10, a slice thickness of 4 mm. For *T*_2_-weighted MR imaging, TR/TE = 1000/18.125 ms, 256 × 256 matrices, 130 × 130 mm field of view, a slice thickness of 4 mm. All the tests were parallelly carried out for three times. Mouse selection and methods of care, welfare, and killing were authorized by the animal ethics committee of Shanghai Normal University, and were in strict accordance with the policy of the Institutional Animal Care and Use Committee.

## 3. Results and discussion

### 3.1. Characterization of the prepared NPs

**Fig. 1:**
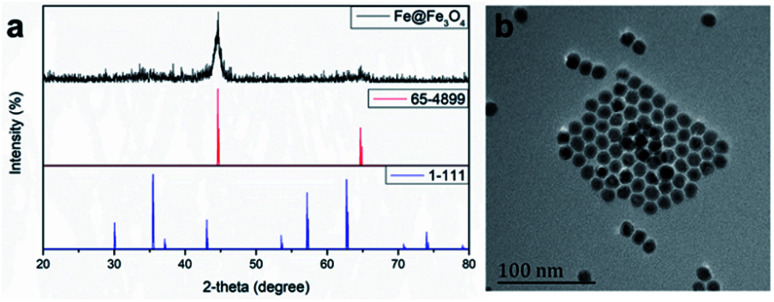
(a) X-ray diffraction pattern and (b) TEM image of Fe@Fe_3_O_4_ nanoparticles.

According the previous reports,^30^ 12 nm Fe@Fe_3_O_4_ nanoparticles were prepared firstly. To verify the crystal structure of the fabricated samples, XRD studies were performed on the obtained dry powder. The two diffraction peaks at 44.6 and 65.2 degree (2*θ*) of the obtained sample are a good match with the (110) and (200) of the body-centered cubic iron (JCPDS card no. 65-4899), respectively (Fig. 1). On the contrary, it is difficult to find the diffraction peak to index to the crystal Fe_3_O_4_ (JCPDS card no. 1-111) due to the extremely poor crystallization of Fe_3_O_4_ in the sample by the natural oxidation method used in the experiment. These results suggest the existence of the body-centered cubic iron, not the crystal Fe_3_O_4_.^31^ In order to demonstrate that the body-centered cubic iron was coated with amorphous Fe_3_O_4_, the TEM was used to further characterize the structure of the prepared sample. The TEM image demonstrate that the obtained sample showed obvious core–shell structures with the shell thickness of ∼2 nm and a high uniformity particle size distribution with a diameter of 12 nm. The similar results with previous literature suggest that the Fe@Fe_3_O_4_ NPs with a core shell structure were prepared successfully.

**Fig. 2:**
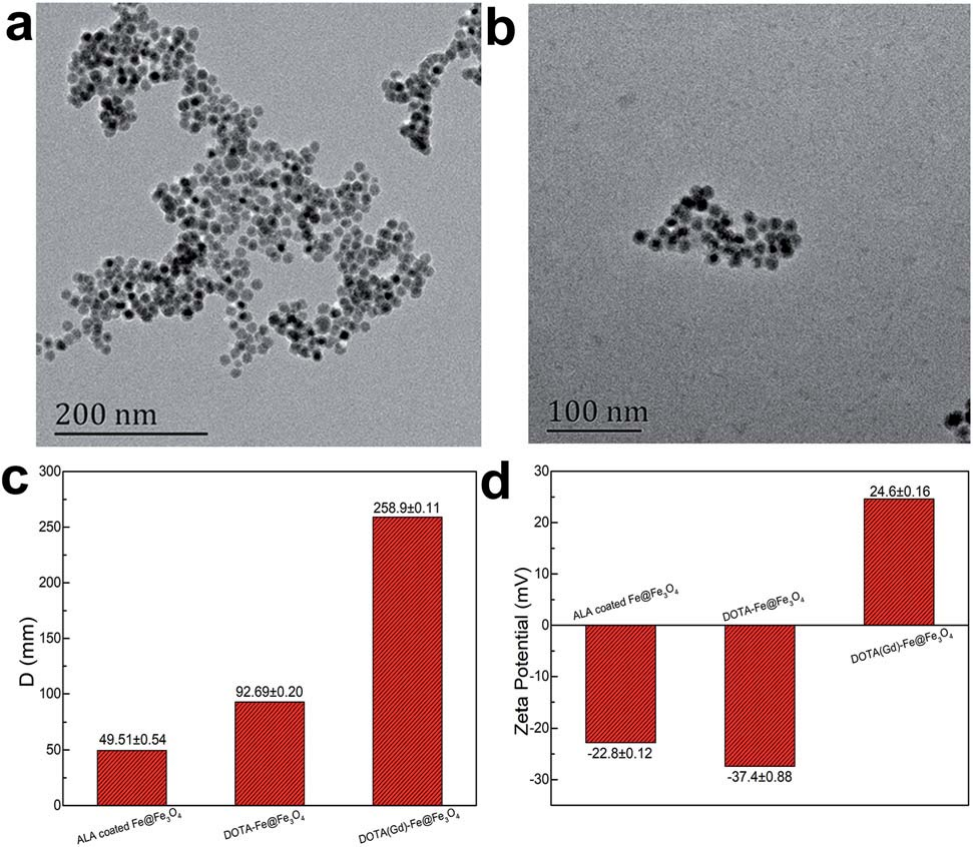
TEM micrograph of (a) ALA coated Fe@Fe_3_O_4_ NPs and (b) DOTA(Gd)-Fe@Fe_3_O_4_ NPs, the comparison images of (c) DLS and (d) zeta potential.

Then, the gadolinium chelate was conjugated to the Fe@Fe_3_O_4_ NPs. In order to obtain the amino group to conjugate the DOTA, the ALA was used to modify the Fe@Fe_3_O_4_ NPs *via* a ligand exchange method at beginning. The results were firstly characterized by the TEM. As shown in Fig. 2, both ALA coated Fe@Fe_3_O_4_ NPs and DOTA(Gd)-Fe@Fe_3_O_4_ NPs are exhibit clearly core–shell structures and a high uniformity particle size distribution with a little bit increased diameter (∼13 and ∼14 nm respectively) after modification, indicating that the modification will not affect structure and size of the Fe@Fe_3_O_4_ NPs. Besides, their DLS results of nanoparticles are shown in Fig. 2c, and it showed that their hydrodynamic diameter were about 49 nm, 92 nm and 258 nm (ESI, Fig. S1†). As a kind of substance with large molecular weight, Gd-DOTA has a strong ability to combine with water, resulting a large size distribution for DOTA(Gd)-Fe@Fe_3_O_4_ NPs. Importantly, the zeta potential of the ALA coated Fe@Fe_3_O_4_ is −22.8 mV. Notably, the surface potential from measurement of DOTA-Fe@Fe_3_O_4_ and DOTA(Gd)-Fe@Fe_3_O_4_ is −37.4 mV and 24.6 mV respectively (Fig. 2d, ESI,† Fig. S2†). The great change of the surface potential caused by the follow reasons. For the ALA coated Fe@Fe_3_O_4_, the observed potential is negative, which is due to the residual phosphate group in the solution is much than the amino group. After grafting with DOTA, the more negative potential can be attributed to the lot of carboxyl groups of DOTA. Once the DOTA(Gd)-Fe@Fe_3_O_4_ formed, the surface potential changed from negative to positive. The result of which advocate the fact that Gd(iii) ions is coordination with the carboxylate groups of DOTA ligand. The great change of the surface potential also can demonstrate that the DOTA(Gd)-Fe@Fe_3_O_4_ was prepared successfully. In order to confirm the elements content of compound, the obtained nanoparticles were determined by ICP assay. According to the results of three parallel tests, the ratio of Fe to Gd is ∼3 : 1.

**Fig. 3:**
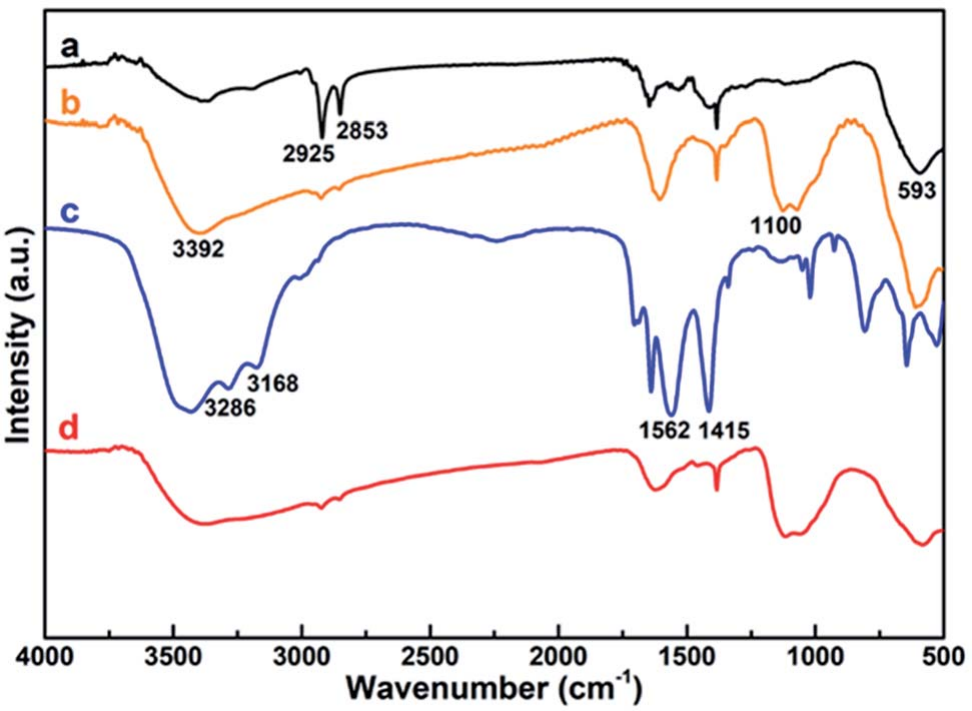
FTIR spectroscopy of (a) Fe@Fe_3_O_4_, (b) ALA coated Fe@Fe_3_O_4_, (c) DOTA-Fe@Fe_3_O_4_ and (d) DOTA(Gd)-Fe@Fe_3_O_4_ NPs.

FTIR was utilized to further characterize the surface ligand of all the obtained NPs, including Fe@Fe_3_O_4_, ALA coated Fe@Fe_3_O_4_, DOTA-Fe@Fe_3_O_4_ and DOTA(Gd)-Fe@Fe_3_O_4_ (Fig. 3). Several characteristic peaks (Fig. 3, origin line a) were observed for Fe@Fe_3_O_4_: 593 cm^−1^ (Fe–O stretch), 2925 cm^−1^ and 2853 cm^−1^ (–CH_2_ vibrations) similar with previous ref. 32 and 33. After coated by the ALA, the spectra showed a significantly band of phosphonate resonance in 1100 cm^−1^ (Fig. 3, origin line b), suggesting the success of ligand exchange. After the attachment of DOTA ligands, two disubstituted amides peaks (3286 cm^−1^, 3168 cm^−1^), a monosubstituted amides peak (1562 cm^−1^) and a strong ester group stretch vibrations (1415 cm^−1^) were observed (Fig. 3, origin line c). When it comes to the DOTA(Gd)-Fe@Fe_3_O_4_ NPs, the vibration absorption peak of the carboxyl group abruptly widens, which should be attributed to the coordination of Gd^3+^ and water.^34^ All of the above results demonstrated that the three-step reaction is successful under the reaction conditions.

### 3.2. Magnetic measurement

**Fig. 4:**
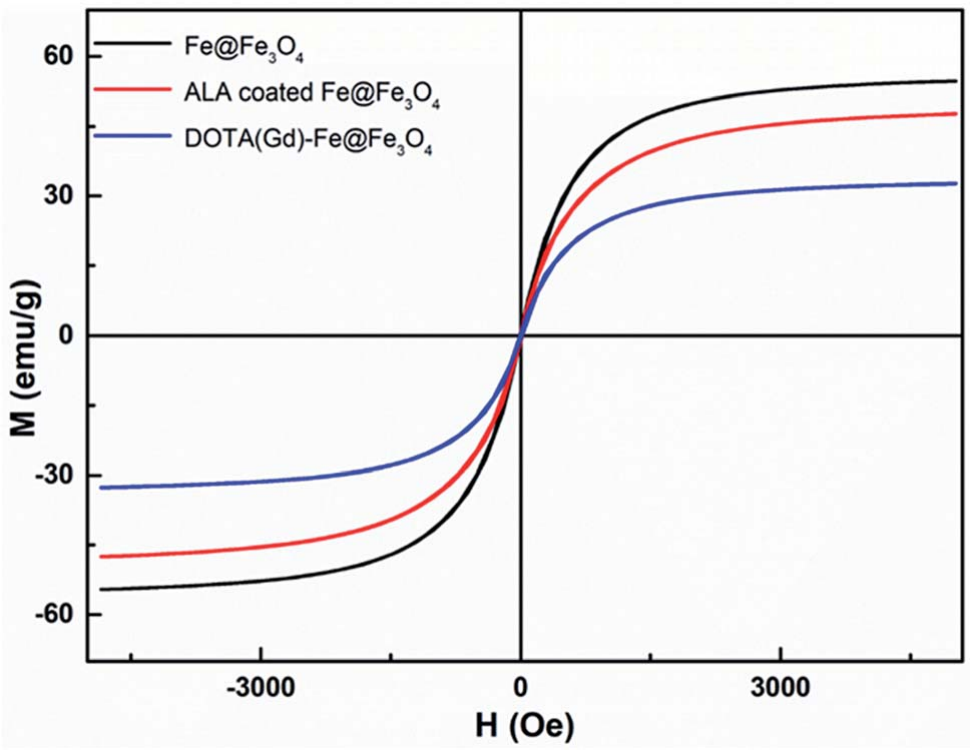
Magnetization curves of bcc-Fe/Fe_3_O_4_, ALA coated Fe@Fe_3_O_4_, DOTA(Gd)-Fe@Fe_3_O_4_.

The magnetic properties of bcc-Fe/Fe_3_O_4_, ALA coated Fe@Fe_3_O_4_, DOTA(Gd)-Fe@Fe_3_O_4_ was investigated by a SQUID magnetometer at room temperature (300 K) (Fig. 4). The results of hysteresis loops indicated that all the nanoparticles exhibit typically paramagnetic behaviors without magnetic hysteresis. The saturation magnetization (*M*_s_) values decrease with the gradual modification of materials. The obtained Fe@Fe_3_O_4_ NPs exhibit strong paramagnetic behaviors with *M*_s_ value of 55 emu g^−1^, which is less than our previous results (∼100 emu g^−1^). The decreased *M*_s_ value can be assigned to more thickness of the Fe_3_O_4_ shell. Once the ALA was utilized to decorate the obtained Fe@Fe_3_O_4_ NPs, the *M*_s_ value reduced to 48 emu g^−1^. The reason for the decrease in *M*_s_ value of the NPs may be the phosphate disodium salt shell coating, which decreased magnetic anisotropy. Moreover, as for the DOTA(Gd)-Fe@Fe_3_O_4_ NPs, the *M*_s_ value decreased to 32 emu g^−1^ which may be because of the content significantly diminishment of iron oxide in the NPs.^35^ The saturation magnetization calculated by the terms of per gram Fe (ESI, Fig. S3†) suggests that the *M*_s_ value increased after conjugating DOTA(Gd) on Fe@Fe_3_O_4_, which is similar with the literature.^28^ Nevertheless, Gd ions can realize the spin order at the same direction with the effect of paramagnetic Fe@Fe_3_O_4_ NPs when exert an external magnetic field. Certainly, the aforementioned changes lead to form a local magnetic field. Hence, the relaxivity of Gd ions was augmented and the homogeneity of magnetic field for *T*_2_ CA was broke as well, and that is why it is always accompanied by improve the *T*_1_ or *T*_2_ contrast effect.^36^ Therefore, we consider that Fe@Fe_3_O_4_@ALA-DOTA(Gd) NPs can show excellent *T*_1_–*T*_2_ dual-modal ability.

**Fig. 5:**
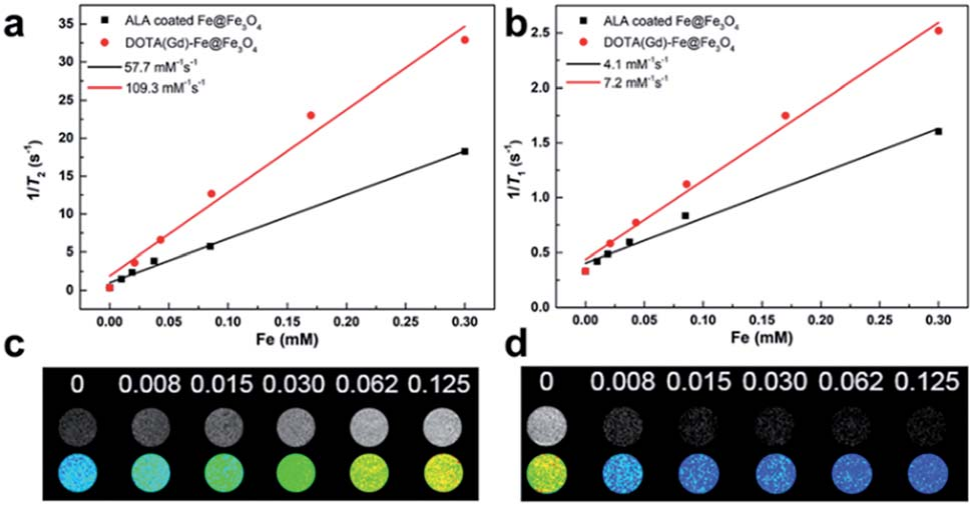
(a) *r*_1_ and (b) *r*_2_ relaxivity values of ALA coated Fe@Fe_3_O_4_ and DOTA(Gd)-Fe@Fe_3_O_4_ NPs; (c) *T*_1_-weighted and (d) *T*_2_-weighted images of DOTA(Gd)-Fe@Fe_3_O_4_ NPs in solution at 0.5 T MR system.

To further provide more evidences to support the capability of dual mode *T*_1_- and *T*_2_-weighted contrast, a 0.5 T MRI scanner was performed to detect the relaxivity of the ALA coated Fe@Fe_3_O_4_ and DOTA(Gd)-Fe@Fe_3_O_4_ NPs in aqueous media. The value of *r*_1_ and *r*_2_ were determined by analyzing the fitted curves for the reciprocal of relaxation time to the Fe concentration. As shown in the Fig. 5a and b, the *r*_1_ relaxivity values of ALA coated Fe@Fe_3_O_4_ and DOTA(Gd)-Fe@Fe_3_O_4_ are 4.1 and 7.2 mM^−1^ s^−1^, while the *r*_2_ relaxivity values are 57.7 and 109.4, respectively. Simultaneously, in order to evaluate the effect of Gd element in NPs, we also made a plot of 1/*T*_1_ against concentration of Gd ion. As shown in the picture (ESI, Fig. S4†), the *r*_1_ of DOTA(Gd)-Fe@Fe_3_O_4_ NPs is much higher than DOTA(Gd). The results show that the DOTA(Gd)-Fe@Fe_3_O_4_ exhibit better relaxivity both for *r*_1_ and *r*_2_, which is benefit for MRI. To now, the highest *r* value for dual mode MR CAs is 836.7 ± 51.1 and 31.6 ± 2.6 for *r*_2_ and *r*_1_ respectively, which is obtained by Long *et al.*^28^ at 1.47 T for DOTA conjugated Fe_3_O_4_. Compared with these reported highest values, the *r* value of DOTA(Gd)-Fe@Fe_3_O_4_ is much lower. However, both of the *r*_2_ and *r*_1_ of DOTA(Gd)-Fe@Fe_3_O_4_ are higher than that of the Fe@Fe_3_O_4_ and DOTA(Gd) alone. These increased *r*_2_ and *r*_1_ are assigned to an alignment of the electronic spins of the paramagnetic ion by the induced magnetic field generated by the superparamagnetic particles.^28^ These results suggest that the prepared DOTA(Gd)-Fe@Fe_3_O_4_ shows great potential for MRI contrast.

Then, the *T*_1_-and *T*_2_-Weighted images of the DOTA(Gd)-Fe@Fe_3_O_4_ was carried out at different Fe concentrations (0 to 0.125 mM) and presented in the Fig. 5c and d. With increasing molar concentration of Fe, the *T*_1_ signal became much brighter, while the *T*_2_ MR contrast became darker, indicating that the DOTA(Gd)-Fe@Fe_3_O_4_ NPs could be utilized as a dual-mode MRI CA. Above all, these results show that the DOTA(Gd)-Fe@Fe_3_O_4_ NPs will exhibit excellent MR signal both for *T*_1_ and *T*_2_.

### 3.3. Cytotoxicity assay

**Fig. 6:**
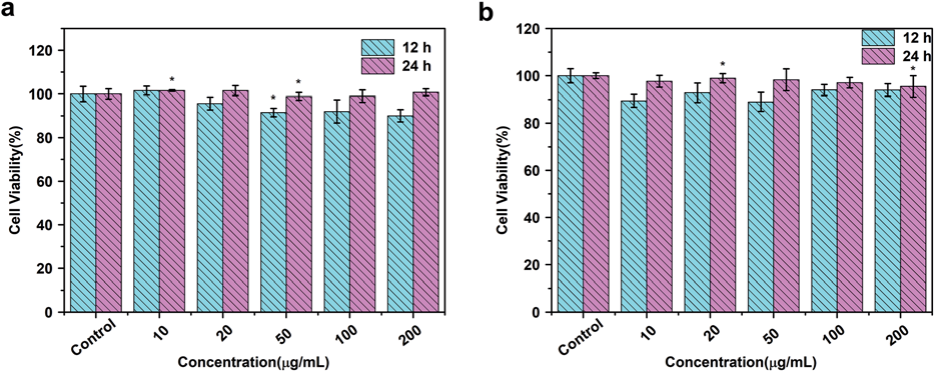
MTT results of (a) 4T1 and (b) HUVEC cells incubated with DOTA(Gd)-Fe@Fe_3_O_4_ NPs in different concentrations for 12 h and 24 h at 37 °C (*n* = 3, *p < 0.05).

To assess the cytotoxic potential effects of DOTA(Gd)-Fe@Fe_3_O_4_ NPs *in vitro* and *in vivo* applications, the particles was evaluated *via* an MTT [3-(4,5-dimethylthiazol-2-yl)-2,5-diphenyltetrazolium bromide] assay for two cell lines, 4T1 (a mouse breast cancer cell lines) and HUVEC (a human umbilical vein endothelial cell lines) respectively. As displayed in Fig. 6, the cells that incubated with the NPs (10, 20, 50, 100, 200 μg mL^−1^) compared to the contrasts hardly show the remarkable difference even for 24 h, which proved the favorable viability of cells. Furthermore, what counts is that the cell viability still remained above 90% when the concentration reaches to 200 μg mL^−1^. Above all, the high cell viability indicates that DOTA(Gd)-Fe@Fe_3_O_4_ NPs exhibit excellent biocompatibility within the experimental concentration (ESI, Fig. S5†). These results establish the foundation of NPs for further biological applications.

### 3.4. Dual *T*_1_–*T*_2_ MR imaging in cells

**Fig. 7:**
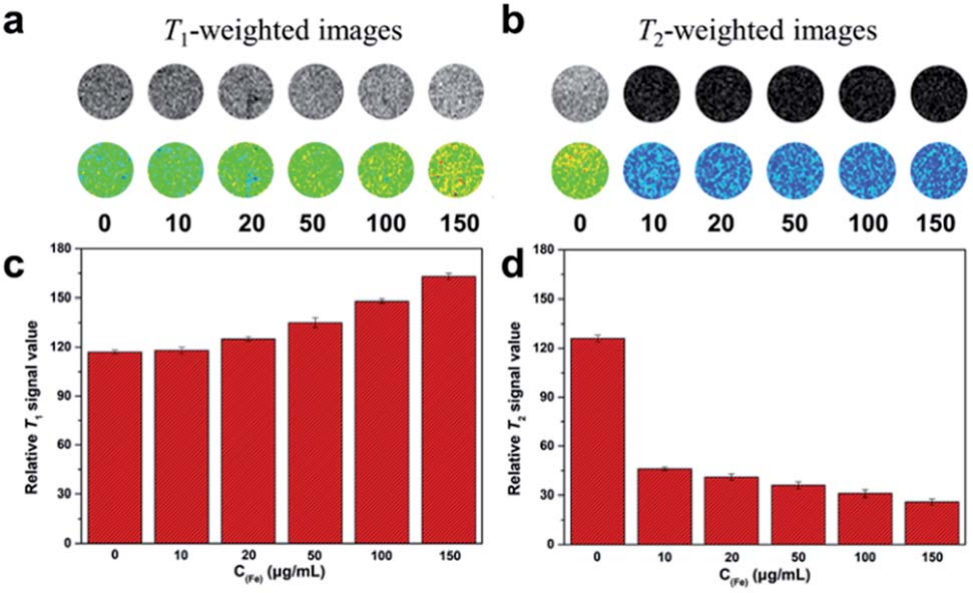
(a) *T*_1_- and (b) *T*_2_-weighted MR imaging of 4T1 cells with various incubate concentration of DOTA(Gd)-Fe@Fe_3_O_4_ NPs. (c) and (d) Signal intensity analysis for MR imaging.

To validate the dual-mode MRI performance of the DOTA(Gd)-Fe@Fe_3_O_4_ NPs, various concentrations samples from 10 to 150 μg mL^−1^ were utilized to incubate the 4T1 cells for 4 h. The *T*_1_- and *T*_2_-weighted MR images and the corresponding signal value of the cells were measured. As displayed in Fig. 7, with DOTA(Gd)-Fe@Fe_3_O_4_ NPs concentration increasing in 4T1 cells, as far as we can see, the positive contrast of *T*_1_-weighted MR images demonstrated an obvious enhancement. Similarly, the 4T1 cells cultivated in particles exhibited a marked negative signal drop with dose-dependent. On the other hand, this point was confirmed *via* collecting the signal intensity changes by quantitative analysis as well. It is worth nothing that DOTA(Gd)-Fe@Fe_3_O_4_ NPs (150 μg mL^−1^) enhance the signal value of *T*_1_ by ∼39.87% compared to the blank group. Simultaneously, the MR signal intensity of *T*_2_ decreased by ∼63.49% after incubation with DOTA(Gd)-Fe@Fe_3_O_4_ NPs even at the lowest concentration. This indicates that the DOTA(Gd)-Fe@Fe_3_O_4_ NPs were successfully taken up by 4T1 cells, which indicated that the dipolar interaction of protons and the magnetic moments in the water and cell media. Besides, the MR contrast on both longitudinal (*T*_1_) and transverse (*T*_2_) proton relaxation time sequences was generated. These results demonstrate that the DOTA(Gd)-Fe@Fe_3_O_4_ NPs can be utilized as a platform for dual *T*_1_–*T*_2_ MRI CAs *in vitro*.

### 3.5. MRI *in vivo*

**Fig. 8:**
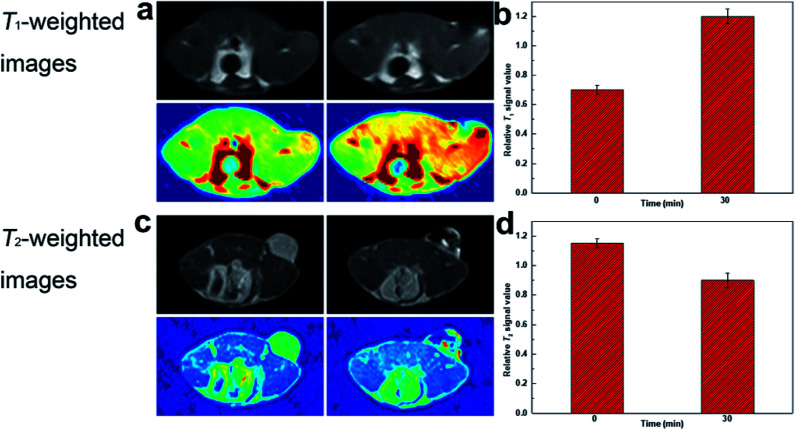
(a) *T*_1_-weighted and (c) *T*_2_-weighted MR images of tumor injected with DOTA(Gd)-Fe@Fe_3_O_4_ NPs; (b) and (d) relative signal intensity for *T*_1_- and *T*_2_-weighted MR images in tumor.

The feasibility of DOTA(Gd)-Fe@Fe_3_O_4_ NPs *in vivo* MRI for tumor was further captured by means of 4T1 murine tumor model. In order to realize the test *in vivo*, the mice were performed by DOTA(Gd)-Fe@Fe_3_O_4_ NPs (50 μL 0.2 mg mL^−1^, 2 mg kg^−1^ of mouse bodyweight) PBS dispersion *via* intratumor injection on a MR imaging scanner. Fig. 8 showed the *T*_1_- and *T*_2_-weighted images of tumor acquired before and 0.5 h post injection images, respectively. Through the comparison of these images, *T*_1_-weighted MR images showed obvious brighter signal. At the same time, *T*_2_-weighted MR images signal at 30 min become darker. This phenomenon is presumably because of the fact that the rapid diffusion of reagent in the tumor site. Furthermore, the *T*_1_ MR images signal intensity was increased by 70% when compared with pre-injection in tumor. While the *T*_2_ MR images signal intensity also exhibited obvious changes, it was decreased by 20% after 30 min post injection. These results declared that DOTA(Gd)-Fe@Fe_3_O_4_ NPs can exhibit positive *T*_1_ or negative *T*_2_ contrast enhancement for tumor MR images simultaneously *via* selecting *r*_1_ or *r*_2_ relaxivities. Therefore, the developed multifunctional DOTA(Gd)-Fe@Fe_3_O_4_ NPs are quite fit for a platform to dual *T*_1_–*T*_2_ MR imaging of tumors.

## 4. Conclusions

In summary, diagnosis is of necessity in clinical application and plays an important role in treatment to some extent. In this work, we successfully manufactured multifunctional Gd-labeled Fe@Fe_3_O_4_ NPs that might act as a dual *T*_1_–*T*_2_ MR CA to reciprocally enhance images of cancer cells both *in vitro* and *in vivo*. These promising NPs have a good biocompatibility and show a high cell viability even at the largest concentration of 200 μg mL^−1^. The accuracy of diagnosis is significantly improved both in positive *T*_1_ and negative *T*_2_ enhancement. The low toxicity and excellent contrast of the developed dual model MRI agents give it a bright future in the clinical application for tumor diagnosis.

## Conflicts of interest

There are no conflicts to declare.

## Supplementary Material

RA-008-C8RA04530E-s001
