# Supplementary material for: Gadolinium-labelled iron/iron oxide core/shell nanoparticles as T1–T2 contrast agent for magnetic resonance imaging
Source: RSC Adv. 2018 Jul 26;8(47):26764–70. doi: 10.1039/c8ra04530e (PMC9083088; doi:10.1039/c8ra04530e)
Supplement: RA-008-C8RA04530E-s001 [file RA-008-C8RA04530E-s001.pdf]

Electronic Supplementary Material (ESI) for RSC Advances.
This journal is © The Royal Society of Chemistry 2018

Supporting Information

Gadolinium-labelled iron/iron oxide core/shell nanoparticles as T_1-T_2 Contrast Agent for Magnetic Resonance Imaging

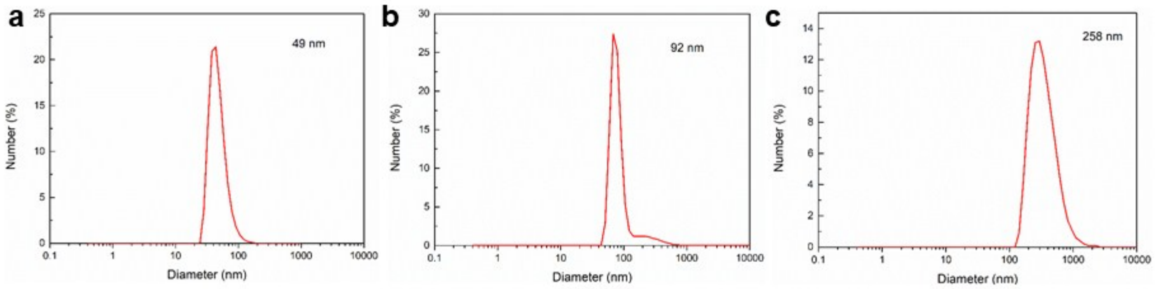

Figure S1. The hydrodynamic diameter of a) ALA coated $Fe@Fe_3O_4$, b) DOTA-$Fe@Fe_3O_4$ and c) DOTA(Gd)-$Fe@Fe_3O_4$ NPs, respectively.

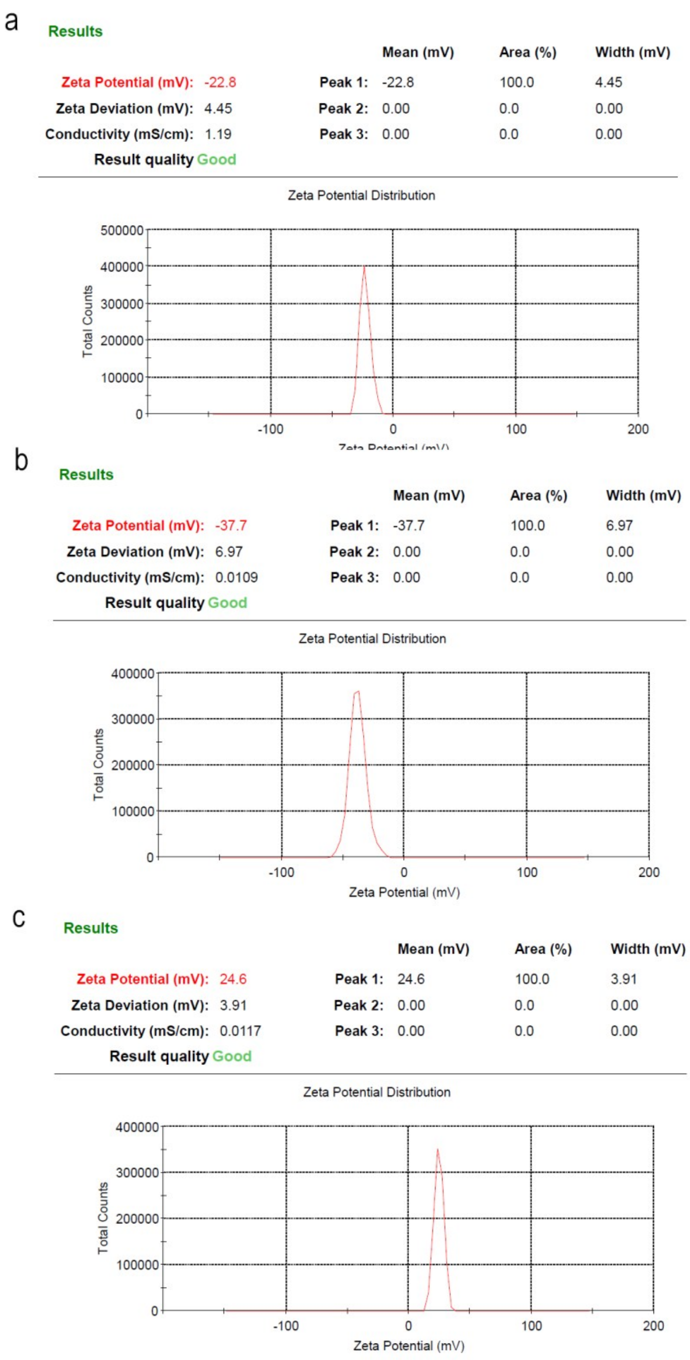

Figure S2. Zeta potentials of a) ALA coated Fe@Fe_3O_4, b) DOTA-Fe@Fe_3O_4 and c) DOTA(Gd)-Fe@Fe_3O_4 NPs, respectively.

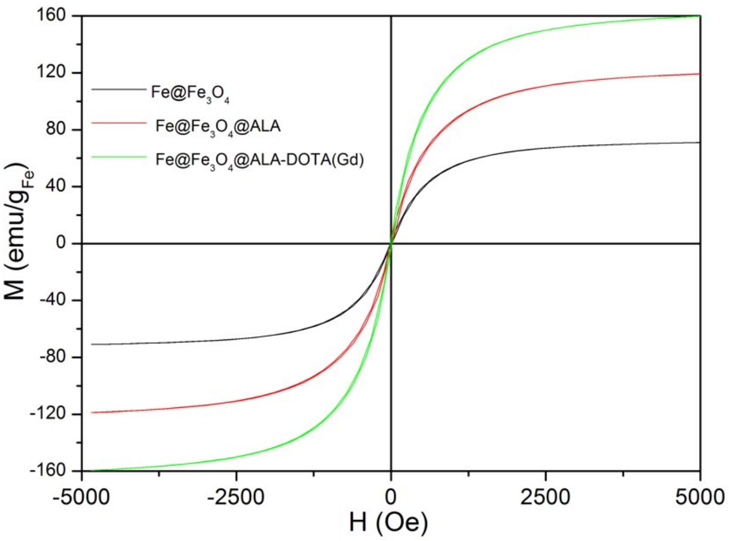

Figure S3. Magnetization curves of bcc-Fe/Fe_3O_4 (black line), ALA coated Fe@Fe_3O_4 (red line), DOTA(Gd)-Fe@Fe_3O_4 (green line) based on the terms of per gram Fe.

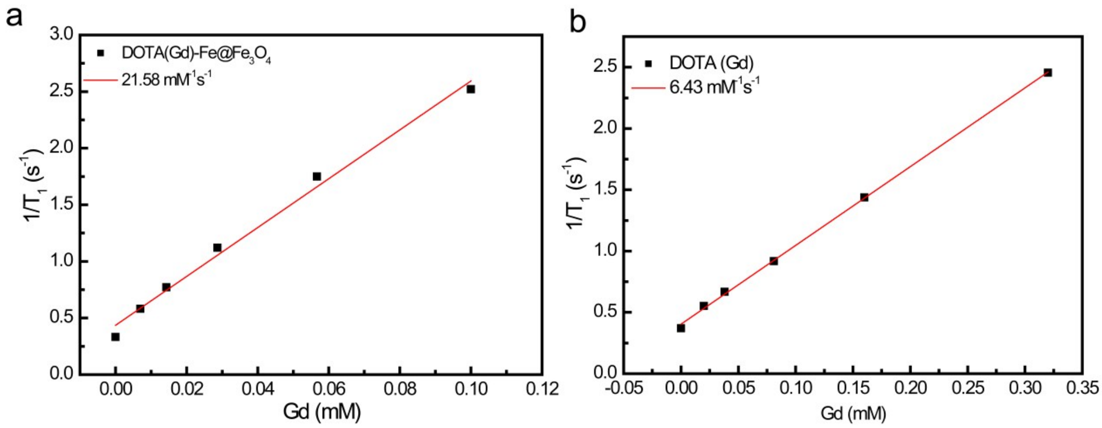

Figure S4. r_1 relaxation rates of a) DOTA(Gd)-Fe@Fe_3O_4 NPs and b) DOTA(Gd) measured at various Gd concentrations with a 0.5 T system.

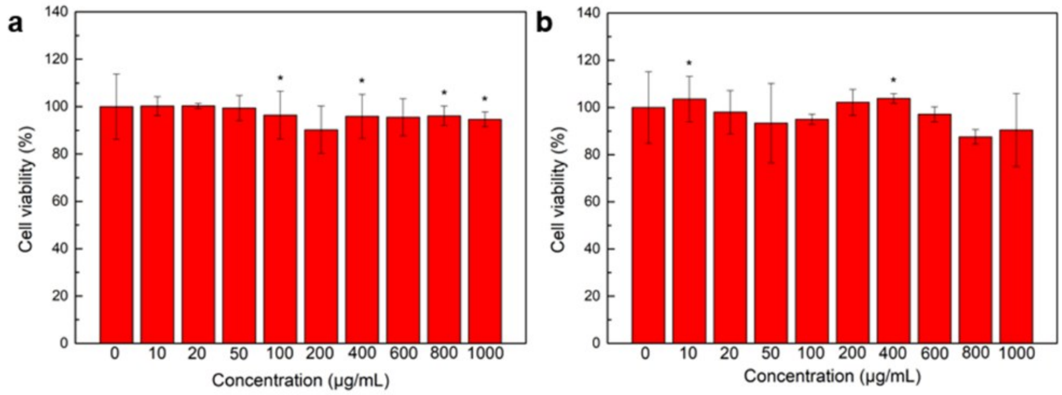

Figure S5. The viability of a) 4T1 and b) HUVEC cell lines incubated with DOTA(Gd)-Fe@Fe_3O_4 NPs with different concentrations for 24 h at 37 ^{o}C, respectively(*$p < 0.05$, $n = 3$).
